# The Development and Validation of a Simple HPLC-UV Method for the Determination of Vancomycin Concentration in Human Plasma and Application in Critically Ill Patients

**DOI:** 10.3390/molecules30051062

**Published:** 2025-02-26

**Authors:** Asma Aboelezz, Novel Solomon Tesfamariam, Maged Kharouba, Tamara Gligoric, Sherif Hanafy Mahmoud

**Affiliations:** 1 Faculty of Pharmacy and Pharmaceutical Sciences, University of Alberta, Edmonton, AB T6G 2E1, Canada; aboelezz@ualberta.ca (A.A.); mkharoub@ualberta.ca (M.K.); tgligori@ualberta.ca (T.G.); 2 Department of Pharmacy, Uppsala University, SE 751 23 Uppsala, Sweden; novelsolomon.tesfamariam.5260@student.uu.se

**Keywords:** vancomycin, HPLC, augmented renal clearance, chromatography

## Abstract

Vancomycin is an antimicrobial agent that exhibits high efficacy against Gram-positive bacteria. The importance of therapeutic drug monitoring (TDM) for vancomycin has been substantiated in specific patient cohorts, underscoring the significance of determining vancomycin plasma levels. This study presents the development and validation of a simple, reproducible, and practical approach for quantifying vancomycin levels in human plasma samples through high-performance liquid chromatography (HPLC). Deproteinization of plasma samples (0.3 mL) was achieved using 10% perchloric acid. The chromatographic separation was achieved using a C18 column. The mobile phase, consisting of phosphate buffer and acetonitrile (90:10, *v*/*v*), was run at a flow rate of 1 mL/min. Ultraviolet detection was conducted at a wavelength of 192 nm and the method was linear in the range of 4.5–80 mg/L (r^2^ > 0.99). Inter- and intra-day assay precision and accuracy were determined to be within the acceptable range. The run time was noted to be 10 min. This method was evaluated using different greenness tools, which indicated that the method is environmentally friendly. Our method was effectively applied to analyze vancomycin concentrations in critically ill patients. Thus, our approach has the potential for practical implementation in routine TDM procedures.

## 1. Introduction

Vancomycin is an antimicrobial agent belonging to the glycopeptide family and was first isolated in 1957 from *Streptomyces orientalis* [1]. Vancomycin is known to be highly effective against Gram-positive bacteria, mainly by inhibiting the bacterial cell wall synthesis [1]. It is also the preferred agent for managing Gram-positive bacterial infections in patients allergic to beta-lactam antimicrobials. Vancomycin is a tricyclic glycopeptide and its molecular mass is about 1500 Da. Since it has negligible oral bioavailability, vancomycin should be administered intravenously (IV) for managing systemic infections [2]. Monitoring the area under the concentration–time curve over the minimum inhibitory concentration (AUC/MIC) is recommended to ensure successful treatment outcomes; however, in clinical practice, obtaining the AUC is often not practical and monitoring the trough concentrations of vancomycin could serve as an alternative for AUC/MIC. For vancomycin to be effective in treating Gram-positive bacteria, AUC/MIC should be ≥400, or trough concentration should fall within the range of 10–20 mg/L [3,4]. Concentrations exceeding the range can be toxic (nephrotoxicity and ototoxicity), while lower values may result in treatment failure; therefore, TDM is recommended to optimize vancomycin therapy. Vancomycin is mainly eliminated through the kidneys and so conditions such as augmented renal clearance (enhanced kidney function, CrCl ≥ 130 mL/min/1.73 m^2^) could affect its plasma concentrations [5]. TDM involves quantitatively analyzing the drug’s concentration in either plasma, serum, or whole blood to ensure it falls within the reference range.

Among the various methods developed for the quantification of vancomycin in biological fluids, immuno-enzymatic techniques and chromatographic methods stand out as the most significant. Although immunoassay techniques are commonly employed in clinical settings because of their simplicity and practicality, these methods measure vancomycin within the reference concentration range [6,7]. When low concentrations of vancomycin or a small amount of samples are anticipated, a more sensitive method, such as high-performance liquid chromatography (HPLC), becomes more appropriate [7].

Ensuring that the method is environmentally friendly using various tools to assess its greenness is of great importance. However, few studies have evaluated the environmental impact of the methods used to analyze vancomycin in biological samples [8]. There are various tools that are used to assess the greenness of the analytical methods that focus on the amounts and types of solvents used, energy consumption, and cost efficiency. Several tools, such as the Analytical method GREEnness score (AMGS) [9], Analytical eco-scale [10], and Analytical GREEness (AGREE) [11], evaluate the impact that analytical methods could have on the environment. These tools provide a score that determines the greenness of the analytical methods.

The aim of this study was the development and validation of a sensitive, selective, rapid, simple, and green HPLC method with UV detection for quantifying vancomycin in human plasma. The advantages of our method compared to others are it utilizes a single simple step of deproteinization using diluted perchloric acid (10%), it has a relatively short run time (less than 10 min), and it is environmentally friendly (green method). Additionally, we applied the method on human samples obtained from critically ill patients treated with vancomycin.

## 2. Results

### 2.1. Optimization of Chromatographic Conditions and Extraction Methods

The development and optimization of the method involved exploring various chromatographic conditions. The elution of vancomycin is affected by the pH of the mobile phase, given that vancomycin is an amphoteric molecule containing both amino and carboxyl groups, which can be ionized under both low and high pH conditions. Therefore, the pH of the mobile phase greatly affects the retention time of vancomycin. Different solvents, including water, methanol, acetonitrile (ACN), mixtures of methanol/ACN/water, and acidic conditions, with the addition of 0.1% Trifluoroacetic acid (TFA) in water or phosphate buffer, were investigated. Mobile-phase compositions of phosphate buffer–ACN (90:10, 88:12, 92:08, and 94:06), different flow rates ranging from 0.6 to 1.2 mL/min, and different wavelengths (192, 205, 215, 220, 254, and 270 nm) were examined. Ultimately, the optimal results were obtained using a low-pH (2.8) phosphate buffer with ACN (90:10 *v*/*v*, respectively) at a flow rate of 1 mL/min and a detection wavelength of 192 nm. The phosphate buffer was prepared using a 0.1 M phosphoric acid mixed with monobasic sodium monophosphate. Since the method was designed for pharmacokinetic (PK) studies and TDM of vancomycin, we minimized the volume of plasma sample to just 0.3 mL. This reduction aimed to reduce the amount of plasma samples needed from patients for analysis. Furthermore, the choice of deproteinization method with 10% perchloric acid was then chosen based on achieving the highest recovery results.

### 2.2. Method Validation

#### 2.2.1. Linearity and Sensitivity

**Figure 1:**
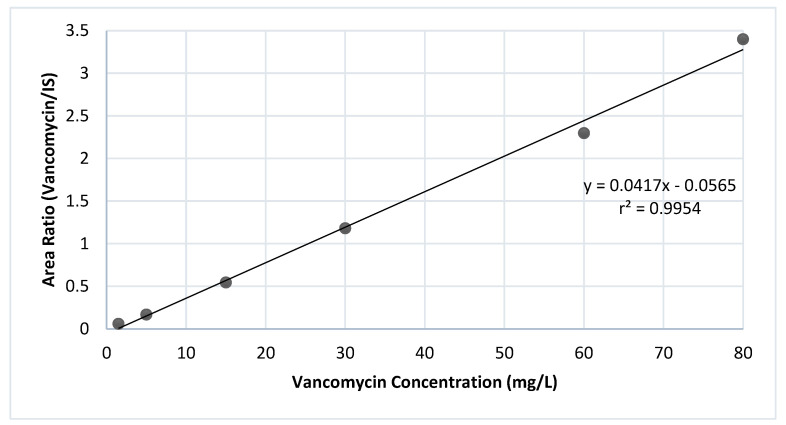
Calibration curve demonstrating the relationship between vancomycin concentration and peak area.

The linearity of the calibration curve was confirmed in the five replicates over a range of 4.5–80 mg/L (r^2^ ≥ 0.99) (Figure 1). The lower limit of quantification (LLOQ) of vancomycin that gave acceptable accuracy and precision was 4.5 mg/L.

#### 2.2.2. Accuracy and Precision

The developed method showed good accuracy and precision (Table 1 and Supplementary Table S1), with an intra-day percentage error and coefficient of variation (CV %) (*n* = 5) of vancomycin within 15%, while the inter-day percentage error and CV were within 8%. These results were within the limits established by the European Medicines Agency (EMA) guideline (15% and 20% for LLOQ).

#### 2.2.3. Carry-Over and Selectivity

**Figure 2:**
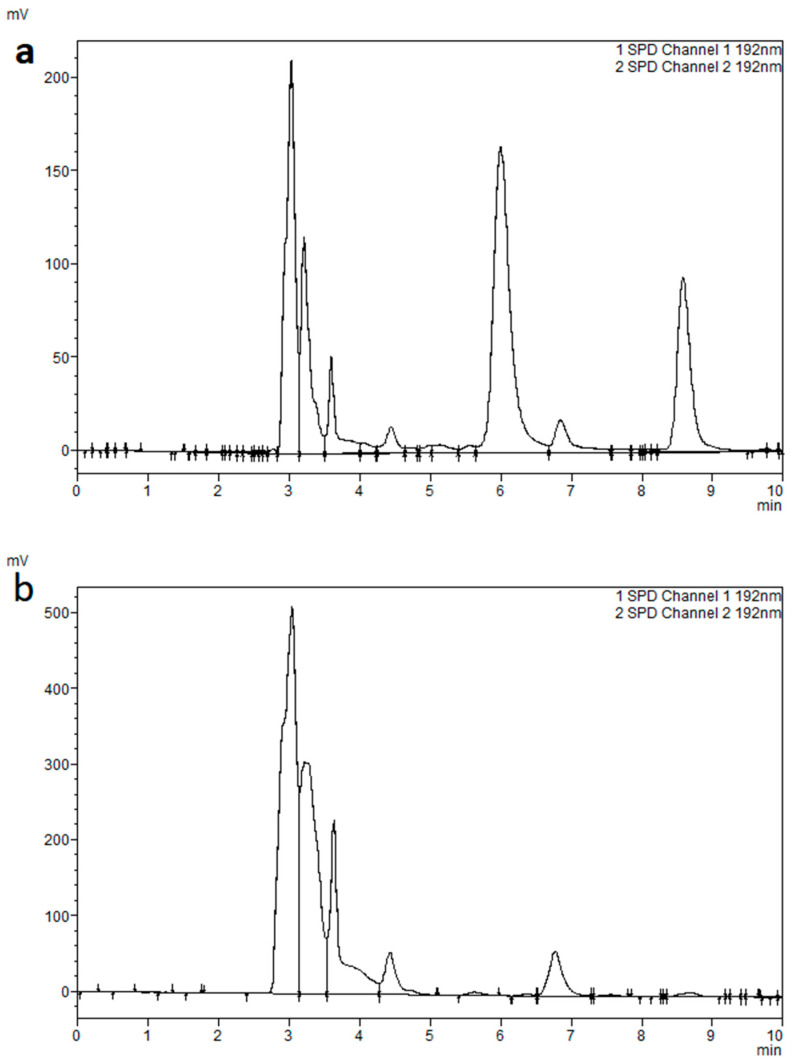
Chromatograms of (**a**) extracted human plasma sample with vancomycin (6.2 min) and IS (8.7 min) and (**b**) extracted blank human plasma sample.

Vancomycin and caffeine were detected at 6.2 and 8.7 min, respectively (Figure 2). To detect carry-over, the upper limit of quantification (ULOQ) was injected followed by an injection of a blank plasma and the chromatogram was clear of any interfering peaks at the vancomycin retention time. Therefore, the method is selective, indicating no interference with plasma ingredients; moreover, no carry-over effects existing after injecting high vancomycin concentrations. However, there was an interfering plasma peak at the caffeine retention time. This peak was within the accepted range, as mentioned by the guidelines.

#### 2.2.4. Recovery

The average percentage recovery of the three quality control (QC) levels ranged from 60.7 to 70.6% (Table 2 and Supplementary Table S2).

#### 2.2.5. Stability

The stability findings (Supplementary Table S3) indicated that vancomycin extracted from human plasma samples exhibits stability on the bench for up to 8 h, displaying acceptable precision (CV %) and accuracy (% error) in the range of 15%. This ensured minimal degradation of vancomycin within the 8 h timeframe, whereas storage for 24 h, 1 week, and 2 weeks revealed substantial degradation, rendering it unsuitable to store extracted samples at room temperature beyond 8 h. Additionally, extracted human plasma samples of vancomycin demonstrated satisfactory precision and accuracy over the entire 2-week period under refrigeration and in −80 °C storage conditions. Conversely, results for vancomycin plasma samples (non-extracted) indicated stability at room temperature for 24 h and for 2 weeks under refrigeration, −20 °C, and −80 °C storage environments. Moreover, following triple freeze and thaw cycles, the precision and accuracy of vancomycin concentrations remained within 15% of the expected concentrations.

### 2.3. Greenness Assessment

Green analytical chemistry aims to develop methods that minimize environmental impact and promote sustainability. We used AMGS [9], the Analytical eco-scale [10], and AGREE [11] tools to evaluate the impact that our method could have on the environment. These tools guide the development of environmentally friendly methods without compromising analytical performance. Evaluating the proposed method using AMGS resulted in a score of 171.16. Moreover, using the AGREE tool resulted in a score of 0.67, while the analytical eco-scale gave a value of 82. These values indicate that this method is green and eco-friendly. An AGREE score of more than 0.5 [11], analytical eco-scale score of more than 75 [10], and the lower AMGS [9] indicates the greenness of the method.

### 2.4. Application of the Method

**Figure 3:**
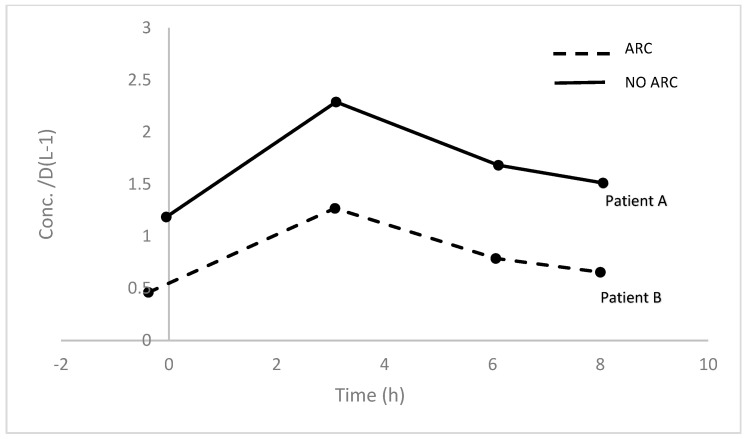
The plasma concentrations (corrected for vancomycin purity) versus time curve of patient A and patient B. Patient A received a dose of 14 mg/kg and patient B received a dose of 21 mg/kg. Despite patient B receiving a higher dose than patient A, patient B had lower plasma concentrations. Such variations could be attributed to the kidney function of the individuals, as patient A had a creatinine clearance (CrCl) of 117 mL/min/1.73 m^2^ and patient B had a CrCl of 159 mL/min/1.73 m^2^. It is observed that patients with augmented renal clearance could experience lower vancomycin concentrations and dosage optimization is required in such a population [5].

The developed method was effectively utilized to measure the concentration of vancomycin in two critically ill patients with varying renal function. A total of eight vancomycin concentrations from two participants were included for analysis, both pre-dose and 1, 4, and 6 h after dose samples were obtained from the patients; the time concentration profile is shown in Figure 3.

## 3. Discussion

Vancomycin, a drug primarily eliminated renally via glomerular filtration, is highly susceptible to fluctuations in renal function and therapeutic drug monitoring is recommended [12]. Herein, we present a straightforward and green analytical method to determine vancomycin concentrations in human plasma.

In developing our analytical method, we undertook a comprehensive optimization process, systematically testing various parameters such as mobile-phase composition, flow rates, and detection wavelengths. Initially, we assessed a buffer-less mobile phase composition. However, this approach resulted in continual shifts in the retention time for vancomycin peaks, attributed to the compound’s high sensitivity to pH variations. To mitigate these retention time shifts, the inclusion of a buffer was deemed necessary. We selected a phosphate buffer with an acidic pH (2.8), which effectively stabilized the retention times. To further refine the mobile phase, we explored different ratios of phosphate buffer to ACN, specifically 90:10, 88:12, 92:08, and 94:06. The optimal ratio of 90:10 yielded the optimal peak separation at 6.2 min for vancomycin without overlapping with the plasma peaks. The chromatogram (Figure 2) shows that this composition produces sharp, well-resolved peaks using this composition. This mobile-phase composition aligns with findings from previous studies [6,8,13,14]. Additionally, we also investigated multiple flow rates in the range of 0.6–1.2 mL/min to determine the optimal conditions for our method. A flow rate of 1 mL/min provided superior elution quality and maintained a reasonable system pressure around 2500 psi, which was within the acceptable operational range of the instrument. Higher flow rates, such as 1.2 mL/min, increased the system pressure, which was considered to be less desirable. On the other hand, flow rates of less than 1 mL/min resulted in an unnecessary increase in the run time. Thus, 1 mL/min was selected for its balance of performance and operational stability. For detection, we tested various UV wavelengths (192, 205, 215, 220, 254, and 270 nm) to identify the most suitable for peak quality, particularly for vancomycin and caffeine. The wavelength of 192 nm produced the best peak quality in terms of peak area, ensuring accurate and reliable quantification of these compounds.

In addition to optimizing our analytical method, we also explored various procedures to efficiently extract vancomycin from human plasma, including liquid–liquid extraction with dichloromethane, hexane, and ethyl acetate, organic solvent precipitation with methanol and acetonitrile, and acid precipitation with TFA, TCA, and perchloric acid. The deproteination method utilizing 10% perchloric acid (100 µL) was chosen based on achieving higher recovery results, making it the preferred choice for our extraction procedure. Moreover, it was preferred due to the rapid deproteinization time, which was around 15 min, compared to other organic solvents that require a longer evaporation time.

Our method validation demonstrated excellent linearity with a correlation coefficient of ≥0.99 with a range of 4.5–80 mg/L, consistent with the findings of Greene et al. and Abu Shandi et al. [13,15]. The method was shown to be accurate and precise, with intra- and inter-day precision and accuracy in the standard limits for both the LLOQ (<20%) and other QC samples (<15%), as per EMA guidelines. The method showed high selectivity, with no interferences observed in the vancomycin peak and no carry-over effect. Sample recovery ranged from 60.7% to 70.6%. Stability tests indicated that plasma samples remained stable for three freeze–thaw cycles and for two weeks under refrigeration and in both −20 °C and −80 °C storage conditions. For extracted plasma samples, stability was maintained for up to 8 h at room temperature to avoid degradation, where stability results were consistent with many published articles [6,15,16,17]. Additionally, compared to previously published studies of analysis of vancomycin, we assessed the environmental friendliness of our method, which was found to be within an acceptable range for method greenness [18].

## 4. Materials and Methods

### 4.1. Chemicals and Reagents

Vancomycin (purity, 91.3%), HPLC-grade acetonitrile, methanol, and other analytical grade reagents (perchloric acid, phosphoric acid, and monobasic sodium monophosphate) were from Sigma-Aldrich (Oakville, ON, Canada). Caffeine, the internal standard (IS), was from Fisher (Ottawa, ON, Canada). Human plasma was purchased from Cedarlane (Burlington, ON, Canada).

### 4.2. Chromatographic Conditions

The chromatographic separation was performed using HPLC-UV (Shimadzu, Kyoto, Japan), which consisted of solvent delivery pumps (LC-20 AT), an SIL-20A autosampler, and a UV-VIS detector (SPD-10A VP). The HPLC chromatographic separation was performed using a reverse-phase Supleco Discovery^®^ C18 column (5 μm, 250 × 4.6 mm) (Supleco Inc., Mississauga, ON, Canada) with a Discovery^®^ C18 Supelguard™ guard column (5 μm, 20 × 4 mm) (Supleco Inc., Mississauga, ON, Canada). LabSolutions Lite^®^ software version 5.93 (Shimadzu, Kyoto, Japan) was used for the acquisition of data and analysis integration. The mobile phase comprised phosphate buffer and ACN (90:10, *v*/*v*). A phosphate buffer with a pH of 2.8 was prepared by combining 175 mL of 0.1 M phosphoric acid with 825 mL of 0.1 M monobasic sodium monophosphate. This mixture resulted in a final volume of 1 L and a pH of 2.8. The final concentration of the phosphate buffer was 0.1 M. Separation was carried out using isocratic elution. The flow rate was set to 1 mL/min. The total run time for sample elution was set to 10 min. The detector was set to a wavelength of 192 nm, with the column and autosampler operated at ambient temperature.

### 4.3. Stock and Working Solutions

The stock solution of vancomycin was prepared by dissolving 6 mg in the mobile phase, making up 6 mL to obtain a concentration of 1 mg/mL. Moreover, stock solution of the IS caffeine was prepared with a concentration of 1 mg/mL. Working solutions of 50 mg/L and 100 mg/L of vancomycin, along with 100 mg/L of the IS, were then prepared by further diluting the stock solutions. Standard solutions for the calibration curve and QC samples were prepared by serial dilution with blank plasma. The concentrations of calibration standards were 80, 60, 30, 15, 10, and 4.5 mg/L, while QC samples included concentrations of 60, 30, 10, and 4.5 mg/L, defined as high-concentration quality control (HQC), medium-concentration quality control (MQC), low-concentration quality control (LQC), and LLOQ, respectively. All solutions were freshly prepared daily at room temperature.

### 4.4. Sample Preparation

Extraction of vancomycin was performed by deproteination of 300 μL plasma sample with 105 μL of 10% perchloric acid. The mixture was vortexed for 40 s and centrifuged at 12,000 rpm for 10 min using an Eppendorf 5424 Microcentrifuge (Eppendorf, Hamburg, Germany). The resultant supernatant was then transferred to a separate HPLC vial with glass inserts. Aliquots of 30 μL was then injected into the HPLC system for analysis. The sample preparation was performed at room temperature and directly injected into the HPLC system.

### 4.5. Method Validation

The developed method was validated according to the EMA 2011 guidelines [19]. The validation process included assessments of linearity, sensitivity, accuracy, precision, carry-over, selectivity, recovery, and stability.

#### 4.5.1. Linearity and Sensitivity

A series of six calibration concentrations spanning from 4.5 to 80 mg/L were constructed to assess linearity through linear regression analysis. Linear regression was conducted by plotting vancomycin concentrations against the peak area ratios (vancomycin/IS). The linearity was then assessed using the coefficient of determination (r2). Vancomycin calibration standard concentrations were then back-calculated using the calibration curve equation. The sensitivity was assessed in terms of the LLOQ, representing the lowest concentration quantifiable with accuracy and precision less than 20%. The selection of the LLOQ was achieved by analyzing replicates of various low concentrations and then measuring the CV% and percentage error, followed by selecting the concentration that gave results within the acceptable range.

#### 4.5.2. Accuracy and Precision

QC sample replicates (*n* = 5) were prepared at four concentration levels, as described previously, over three consecutive days. These samples were analyzed to assess both intra-day and inter-day accuracy and precision. The vancomycin concentrations were determined using a standard calibration curve, which was prepared daily with the samples. Accuracy at each concentration level was assessed by comparing the observed concentration to the nominal concentration, with results expressed as percentage error. Precision was evaluated by calculating CV% for each measurement.

#### 4.5.3. Carry-Over and Selectivity

Carry-over was evaluated by first injecting a high-concentration sample (e.g., ULOQ 80 mg/L), followed by injecting a blank plasma sample. According to the EMA guidelines, the acceptable limit for carry-over was defined as the blank plasma response not exceeding ±20% and ±5% for the peak area response of the vancomycin LLOQ and the IS, respectively. Method selectivity was evaluated by comparing the chromatograms of blank plasma with those from vancomycin and IS plasma samples. For the method to be considered selective, no peaks should be interfering with the vancomycin and IS peaks.

#### 4.5.4. Recovery

The developed method average extraction recovery was determined by injecting three replicates of extracted samples at 3 different QC levels (5, 20 and 50 mg/L). The sample peak areas were compared to peak areas obtained from extracted blank plasma samples spiked with equivalent vancomycin and IS concentrations.

#### 4.5.5. Stability

The method stability was evaluated through a comparison of the vancomycin concentrations from freshly prepared samples (10 and 70 mg/L, *n* = 5) before and after exposure to various conditions in extracted human plasma and in non-extracted human plasma samples. The conditions for stability assessment were designed to simulate the conditions encountered during sample storage, handling, and analysis. The stability of vancomycin in extracted human plasma was evaluated at different temperatures including room temperature, −80 °C, and in a refrigerator (4 °C) over a period of two weeks. Furthermore, the stability of vancomycin in human plasma was investigated at room temperature (24 h), in a refrigerator, and at −20 °C and −80 °C for a duration of 2 weeks. Additionally, we conducted an assessment of freeze–thaw stability involving three cycles, each comprising a 24 h freezing period followed by room temperature thawing, with the cycle being repeated. Acceptability criteria included a stability ratio to reference samples falling within the range of 85% to 115% and a percentage error within 15%.

### 4.6. Greenness Assessment

The greenness of analytical methods was evaluated using three tools: AMGS (2019) [9], Analytical eco-scale (2012) [10], and AGREE (2020) [11].

### 4.7. Method Application

We applied our method in the analysis of vancomycin plasma concentrations obtained from participants enrolled in the Neuro-ARC study conducted by our lab. The neuro-ARC study is a multicenter prospective observational study that aimed to characterize the phenomenon of augmented renal clearance (ARC) in neurocritical care patients admitted with life-threatening neurological injury. Written informed consent was obtained and the study was approved by the Health Research Ethics Board of the University of Alberta. The purity of vancomycin (91.3%) was taken into account for measuring the patients’ vancomycin concentrations.

## 5. Conclusions

The developed study details a comprehensively validated chromatographic HPLC-UV method for accurately quantifying vancomycin in human plasma. Our validated method is characterized by its simplicity, reproducibility, sensitivity, stability, and greenness under various storage conditions. Its simplicity is evident in the straightforward extraction procedure involved. Moreover, our method is capable of quantifying vancomycin concentrations, which could guide the clinical decision of its dosing. Notably, it requires only a limited sample volume to run the analysis. This combination of features makes the analysis cost-effective as well as time-effective, rendering this method well suited for routine TDM and research applications.

## Figures and Tables

**Table 1:** Inter-day and intra-day precision and accuracy for vancomycin human plasma samples.

Nominal Concentration (mg/L) *n* = 5	Mean ± SD (mg/L)	CV%	Error%
Intra-day precision and accuracy ^a^			
4.5 (LLOQ)	4.42 ± 0.3	6.79	1.84
10 (LQC)	9.96 ± 0.35	3.53	0.36
30 (MQC)	28.19 ± 2.36	8.39	6.02
60 (HQC)	60.54 ± 1.81	2.99	0.91
Inter-day precision and accuracy ^b^			
4.5 (LLOQ)	4.83 ± 0.29	6.06	7.36
10 (LQC)	10.37 ± 0.57	5.51	3.71
30 (MQC)	28.24 ± 0.77	2.71	5.85
60 (HQC)	57.27 ± 3.19	5.58	4.53

^a^ Analyzed on same day (intra-day); ^b^ analyzed on three different days (inter-day); CV, coefficient of variation; SD, standard deviation; HQC, higher-quality control sample; MQC, medium-quality control sample; LQC, lower-quality control; LLOQ, lower limit of quantification.

**Table 2:** Mean percent recovery for vancomycin and caffeine ratio.

Vancomycin Concentrations (mg/L) *n* = 3	Vancomycin/IS Recovery (%)
5	70.5
20	64.8
50	60.7

## Data Availability

The raw data supporting the conclusions of this article will be made available by the corresponding author upon request.

## Supplementary Materials

The following supporting information can be downloaded at https://www.mdpi.com/article/10.3390/molecules30051062/s1, Details of the method validation results are provided in Supplementary, Table S1: Accuracy and Precision; Table S2: Recovery; Table S3: Stability.

## Abbreviations

The following abbreviations are used in this manuscript:

MDPI	Multidisciplinary Digital Publishing Institute
IV	Intravenously
AUC/MIC	Area under the concentration–time curve over the minimum inhibitory concentration
CrCl	Creatinine clearance
HPLC	High-performance liquid chromatography
ACN	Acetonitrile
TFA	Trifluoroacetic acid
PK	Pharmacokinetic
LLOQ	Lower limit of quantification
CV	Coefficient of variation
EMA	European Medicines Agency
ULOQ	Upper limit of quantification
QC	Quality control
AMGS	Analytical method GREEnness score
AGREE	Analytical GREEness
IS	Internal standard
HQC	Higher-quality control
MQC	Medium-quality control
LQC	Lower-quality control
r2	coefficient of determination

## References

[1] Rubinstein E., Keynan Y. (2014). Vancomycin revisited–60 years later. Front. Public Health.

[2] Aradhyula S., Manian F.A., Hafidh S.A., Bhutto S.S., Alpert M.A. (2006). Significant absorption of oral vancomycin in a patient with Clostridium difficile colitis and normal renal function. South. Med. J..

[3] Tesfamariam N.S., Aboelezz A., Mahmoud S.H. (2024). The Impact of Augmented Renal Clearance on Vancomycin Pharmacokinetics and Pharmacodynamics in Critically Ill Patients. J. Clin. Med..

[4] Rybak M.J., Le J., Lodise T.P., Levine D.P., Bradley J.S., Liu C., Mueller B.A., Pai M.P., Wong-Beringer A., Rotschafer J.C. (2020). Therapeutic monitoring of vancomycin for serious methicillin-resistant Staphylococcus aureus infections: A revised consensus guideline and review by the American Society of Health-System Pharmacists, the Infectious Diseases Society of America, the Pediatric Infectious Diseases Society, and the Society of Infectious Diseases Pharmacists. Am. J. Health-Syst. Pharm..

[5] Mahmoud S.H., Shen C. (2017). Augmented Renal Clearance in Critical Illness: An Important Consideration in Drug Dosing. Pharmaceutics.

[6] Usman M., Hempel G. (2016). Development and validation of an HPLC method for the determination of vancomycin in human plasma and its comparison with an immunoassay (PETINIA). Springerplus.

[7] Cheng X., Ma J., Su J. (2022). An overview of analytical methodologies for determination of vancomycin in human plasma. Molecules.

[8] Jahan F., Zaman S.-u., Syed M.A., Arshad R., Amjad O., Ali I., Gul R., Shahnaz G., Aamir M.N., Hanif S. (2022). HPLC method development and validation for in vitro and in vivo quantification of vancomycin in rabbit plasma. Pak. J. Pharm. Sci..

[9] Hicks M.B., Farrell W., Aurigemma C., Lehmann L., Weisel L., Nadeau K., Lee H., Moraff C., Wong M.L., Huang Y. (2019). Making the move towards modernized greener separations: Introduction of the analytical method greenness score (AMGS) calculator. Green Chem..

[10] Galuszka A., Konieczka P., Migaszewski Z.M., Namiesnik J. (2012). Analytical Eco-Scale for assessing the greenness of analytical procedures. TrAC-Trend Anal. Chem..

[11] Pena-Pereira F., Wojnowski W., Tobiszewski M. (2020). AGREE-Analytical GREEnness Metric Approach and Software. Anal. Chem..

[12] Ji X.-w., Ji S.-m., He X.-r., Zhu X., Chen R., Lu W. (2018). Influences of renal function descriptors on population pharmacokinetic modeling of vancomycin in Chinese adult patients. Acta Pharmacol. Sin..

[13] Greene S.V., Abdalla T., Morgan S.L., Bryan C.S. (1987). High-performance liquid chromatographic analysis of vancomycin in plasma, bone, atrial appendage tissue and pericardial fluid. J. Chromatogr. B Biomed. Sci. Appl..

[14] Lukša J., Marušič A. (1995). Rapid high-performance liquid chromatographic determination of vancomycin in human plasma. J. Chromatogr. B Biomed. Sci. Appl..

[15] Abu-Shandi K.H. (2009). Determination of vancomycin in human plasma using high-performance liquid chromatography with fluorescence detection. Anal. Bioanal. Chem..

[16] Hagihara M., Sutherland C., Nicolau D.P. (2013). Development of HPLC methods for the determination of vancomycin in human plasma, mouse serum and bronchoalveolar lavage fluid. J. Chromatogr. Sci..

[17] López K.V., Bertoluci D.F., Vicente K.M., Dell’Aquilla A., Santos S.J. (2007). Simultaneous determination of cefepime, vancomycin and imipenem in human plasma of burn patients by high-performance liquid chromatography. J. Chromatogr. B.

[18] Lima T.M., Seba K.S., Goncalves J.C.S., Cardoso F.L.L., Estrela R.C.E. (2018). A Rapid and Simple HPLC Method for Therapeutic Monitoring of Vancomycin. J. Chromatogr. Sci..

[19] Guideline on Bioanalytical Method Validation. https://www.ema.europa.eu/en/documents/scientifc-guideline/guideline-bioanalytical-methodvalidation_en.pdf.

