# Supplementary material for: The Development and Validation of a Simple HPLC-UV Method for the Determination of Vancomycin Concentration in Human Plasma and Application in Critically Ill Patients
Source: Molecules. 2025 Feb 26;30(5):1062. doi: 10.3390/molecules30051062 (PMC11901705; doi:10.3390/molecules30051062)
Supplement: Supplementary file 1 [file molecules-30-01062-s001.zip › molecules-3448355-supplementary.pdf]

The Development and Validation of a Simple HPLC-UV Method for the Determination of Vancomycin Concentration in Human Plasma and Application in Critically Ill Patients

Supplementary Materials: Method Validation results

Table S1. Accuracy and Precision

Accuracy and precision over 3 days

Vancomycin Concentrations (mg/L)	Mean area ratios of vancomycin/IS n=5	Mean back calculated vancomycin concentrations (mg/L)	Standard deviation (SD)	CV %	% Error
**Day 1**					
4.5	0.154	5.055	0.162	3.205	12.333
10	0.232	11.180	0.588	5.259	11.800
30	1.083	27.334	1.620	5.927	8.887
60	2.151	52.942	4.439	8.385	11.763
**Day 2**					
4.5	0.178	5.020	0.296	5.896	11.556
10	0.166	9.968	0.352	3.520	0.320
30	0.944	29.208	1.304	4.465	2.640
60	1.868	58.351	4.025	6.898	2.748
**Day 3**					
4.5	0.205	4.417	0.300	6.792	1.844
10	0.166	9.964	0.352	3.533	0.360
30	1.0515	28.194	2.366	8.392	6.020
60	2.203	60.544	1.812	2.993	0.907

Abbreviations; CV, Coefficient of variation; IS, internal standard; SD, standard deviation.

* Day 3 was selected to represent intra-day precision and accuracy, however, all three days results are within acceptable range.

Table S2. Recovery

Vancomycin concentrations (mg/L)	Mean area ratios of vancomycin/IS n=3	Vancomycin/IS recovery (%)
5 (sample)	0.251	70.506
5 (standard)	0.356	
20 (sample)	0.761	64.766
20 (standard)	1.175	
50 (sample)	1.460	60.707
50 (standard)	2.405	

Abbreviations; IS, internal standard

Table S3. Stability

Extracted plasma stability

3.1 Extracted Plasma stability at room temperature

Vancomycin Concentrations (mg/L)	Mean back calculated Vancomycin Concentrations (mg/L) n=5	Standard Deviation (SD)	CV %	% Error
**At zero hour**				
10	10.050	0.664	6.607	0.500
70	61.570	0.514	0.835	12.043
**At 3 hours**				
10	9.283	0.294	3.167	7.170
70	66.607	0.784	1.375	4.847
**At 8 hours**				
10	10.141	1.722	16.980	1.410
70	77.987	4.486	5.752	11.410
**At 24 hours**				
10	6.792	0.187	2.753	32.080
70	41.340	0.313	0.757	40.943
**1 week**				
10	2.491	0.130	5.219	75.090
70	12.190	0.105	0.861	82.586
**2weeks**				
10	0.722	0.127	17.590	92.780
70	3.647	0.126	3.455	94.790

Abbreviations; CV, Coefficient of variation; SD, standard deviation

3.2 Extracted Plasma stability at fridge temperature

Vancomycin Concentrations (mg/L)	Mean back calculated Vancomycin Concentrations (mg/L) n=5	Standard Deviation (SD)	CV %	% Error
**At zero hour**				
10	10.050	0.664	6.607	0.500
70	61.570	0.514	0.835	12.043
**At 3 hours**				
10	8.720	2.196	25.183	12.800
70	64.073	5.600	8.740	8.467
**At 24 hours**				
10	9.740	0.177	1.817	2.600
70	76.528	3.408	4.453	9.326
**2weeks**				
10	8.307	0.458	5.513	16.930
70	77.022	0.602	0.782	10.031

Abbreviations; CV, Coefficient of variation; SD, standard deviation

3.3 Extracted Plasma stability at -80 temperature

Vancomycin Concentrations (mg/L)	Mean back calculated Vancomycin Concentrations (mg/L) n=5	Standard Deviation	CV %	% Error
**At zero hour**				
10	10.050	0.664	6.607	0.500
70	61.570	0.514	0.835	12.043
**At 24 hours**				
10	9.888	0.419	4.237	1.120
70	82.311	2.447	2.973	17.587
**2weeks**				
10	8.980	0.375	4.176	10.200
70	82.096	1.113	1.356	17.280

Abbreviations; CV, Coefficient of variation; SD, standard deviation

Non-extracted plasm stability

3.4 Plasma stability at room temperature

Vancomycin Concentrations (mg/L)	Mean back calculated Vancomycin Concentrations (mg/L) n=5	Standard Deviation (SD)	CV %	% Error
**At zero hour**				
10	10.050	0.664	6.607	0.500
70	61.570	0.514	0.835	12.043
**At 3 hours**				
10	9.520	0.075	0.788	4.800
70	70.355	0.649	0.922	0.507
**At 24 hours**				
10	9.543	0.133	1.394	4.570
70	67.737	2.442	3.605	3.233

Abbreviations; CV, Coefficient of variation; SD, standard deviation

3.5 Plasma stability at Fridge temperature

Vancomycin Concentrations (mg/L)	Mean back calculated Vancomycin Concentrations (mg/L) n=5	Standard Deviation (SD)	CV %	% Error
**At zero hour**				
10	10.050	0.664	6.607	0.500
70	61.570	0.514	0.835	12.043
**At 3 hours**				
10	9.276	0.328	3.536	7.240
70	75.272	1.645	2.185	7.531
**At 24 hours**				
10	8.860	0.321	3.623	11.400
70	69.461	0.674	0.970	0.770
**2weeks**				
10	8.404	0.150	1.785	15.960
70	69.110	0.838	1.213	1.271

Abbreviations; CV, Coefficient of variation; SD, standard deviation

3.6 Plasma stability at -20 temperature

Vancomycin Concentrations (mg/L)	Mean back calculated Vancomycin Concentrations (mg/L) n=5	Standard Deviation (SD)	CV %	% Error
**At zero hour**				
10	10.050	0.664	6.607	0.500
70	61.570	0.514	0.835	12.043
**2 weeks**				
10	8.179	0.238	2.910	18.210
70	60.940	3.286	5.392	12.943

Abbreviations; CV, Coefficient of variation; SD, standard deviation

3.7 Plasma stability at - 80 temperature

Vancomycin Concentrations (mg/L)	Mean back calculated Vancomycin Concentrations (mg/L) n=5	Standard Deviation (SD)	CV %	% Error
**At zero hour**				
10	10.050	0.664	6.607	0.500
70	61.570	0.514	0.835	12.043
**At 24 hours**				
10	9.282	0.248	2.672	7.180
70	67.964	1.916	2.819	2.909
**2weeks**				
10	8.809	0.238	2.702	11.910
70	69.056	1.538	2.227	1.349

Abbreviations; CV, Coefficient of variation; SD, standard deviation

3.8 Freeze and thaw stability

Vancomycin Concentrations (mg/L)	Mean back calculated Vancomycin Concentrations (mg/L) n=5	Standard Deviation (SD)	CV %	% Error
10	9.289	0.317	3.413	7.110
70	77.933	1.700	2.181	11.333

Abbreviations; CV, Coefficient of variation; SD, standard deviation
